# Abnormal Brain Structure Morphology in Early-Onset Schizophrenia

**DOI:** 10.3389/fpsyt.2022.925204

**Published:** 2022-07-07

**Authors:** Jia Cai, Wei Wei, Liansheng Zhao, Mingli Li, Xiaojing Li, Sugai Liang, Wei Deng, Xiang Dong Du, Qiang Wang, Wan-jun Guo, Xiaohong Ma, Pak C. Sham, Tao Li

**Affiliations:** ^1^Mental Health Center, West China Hospital of Sichuan University, Chengdu, China; ^2^Affiliated Mental Health Center & Hangzhou Seventh People’s Hospital, Zhejiang University School of Medicine, Hangzhou, China; ^3^Suzhou Psychiatry Hospital, Affiliated Guangji Hospital of Soochow University, Suzhou, China; ^4^Department of Psychiatry, Li Ka Shing Faculty of Medicine, The University of Hong Kong, Hong Kong SAR, China; ^5^Center for PanorOmic Sciences, The University of Hong Kong, Hong Kong SAR, China; ^6^State Key Laboratory of Brain and Cognitive Sciences, The University of Hong Kong, Hong Kong SAR, China

**Keywords:** schizophrenia, MRI, early onset, cerebral cortex, gray matter

## Abstract

With less exposure to environmental and medication influences, individuals with early-onset schizophrenia (EOS) may provide valuable evidence to study the pathogenesis and phenotypic pattern of schizophrenia.T1-weighted magnetic resonance images were collected in 60 individuals with EOS and 40 healthy controls. Voxel-based morphometry and surface-based morphometry analyzes were performed. Gray matter volume, cortical thickness and cortical surface area were compared between the EOS and healthy controls and among schizophrenia subgroups (with or without family history of schizophrenia). Compared with healthy controls, the EOS group had reduced gray matter volume in the bilateral middle temporal gyrus and reduced cortical thickness in several brain regions. The sporadic early onset schizophrenia and the familial early onset schizophrenia showed different brain structure morphology. These findings suggest that abnormal brain structure morphology, especially in the temporal and frontal lobes, may be an important pathophysiological feature of EOS.

## Introduction

Schizophrenia is a group of severe psychiatric disorders with unknown etiology. Individuals who are diagnosed with schizophrenia before the age of 18 years are defined as having early-onset schizophrenia (EOS) (1). The prevalence of EOS accounts for approximately 4–5% of all schizophrenia cases (2). EOS shows more severe symptoms, a longer duration of untreated illness, and a poorer response to medication than adult-onset schizophrenia (AOS) (3). With relatively little exposure to the environment and medication, people with EOS may be a source of valuable evidence regarding the pathogenesis and phenotypic pattern of schizophrenia.

In the effort understand the etiology of schizophrenia, there has been increasing focus on structural brain abnormalities in schizophrenia, as evidenced by a large number of magnetic resonance imaging (MRI) studies. There are fewer studies on EOS than on AOS. Previous studies reported decreased grey matter volume (GMV) in the frontal, temporal and parietal lobes in EOS (4, 5). However, some studies found increased GMV in the temporal lobe (6), whereas others did not find any change (7). Abnormal cortical thickness and cortical surface area in people with EOS were also reported (8–10). A cross-sectional study found that the average cortical thickness of the EOS group was significantly thinner (7.5%) than that of healthy controls (HCs) (11). Later studies showed reduced cortical thickness in the frontal and temporal lobes in the EOS group (12, 13). Reduced cortical thickness in the parietal lobe, corpus callosum, hippocampus and posterior central gyrus has also been reported (14, 15). Healthy siblings of those with EOS also showed a pattern of reduced cortical thickness in the frontal, temporal, and parietal lobes (16). Although these are promising findings, the evidence supporting abnormal brain structure morphology in EOS remains equivocal. There are several possible reasons for this. First, most studies included small sample sizes, which limited the statistical power. Second, the diagnosis categories and the age range of participants varied in the studies. For example, some studies included other mental disorders, such as bipolar disorder or schizoaffective disorder, while some studies included subjects with a wide range of ages or included subjects who developed the disorder before the age of 18 years but were adults when participating in the study (17). Third, a relatively long course of illness and antipsychotic medications may affect the structure of the brain (12, 13). Therefore, brain structure morphology in EOS has yet to be confirmed.

Genetic factors play an important role in the pathogenesis of schizophrenia (18). Individuals with familial schizophrenia (FSP) and those with sporadic schizophrenia (SSP) showed different brain structure/functional connectivity, although the findings were inconsistent (19–22). No studies thus far have explored whether the presence of a family history of schizophrenia causes differences in brain structure in EOS. In the study, we conducted an exploratory analysis of this possibility.

To address the above questions, we included a relatively large sample of people with EOS whose average age was 14 years; most of them had received low-dose antipsychotics for less than a week and had a short disease duration, i.e., less than 6 months. Voxel-based morphometry (VBM) is the most commonly used algorithm in the study of GMV (23). As an alternative method, surface-based morphometry (SBM) can detect changes in the cerebral grey matter (GM), and it can also provide an independent definition of GM thinning and regional surface area change (24). In this study, we combined VBM and SBM analyzes to explore the macrostructural changes in the EOS group in a Han Chinese population and to further conduct an exploratory analysis on whether a family history of psychiatric disorder was related to the severity of abnormalities in brain structure morphology.

## Materials and Methods

### Participants

Sixty-six participants with EOS were recruited from inpatient and outpatient psychiatric units at West China Hospital, Sichuan University. Diagnosis was made according to DSM-IV criteria. All participants were interviewed using the Structured Clinical Interview for the DSM-IV (SCID-P). Subjects also underwent further clinical evaluation by using the Positive and Negative Syndrome Scale (PANSS) (25). Six subjects were excluded due to poor-quality MRI scans. The psychiatric history of each subject was reviewed to exclude those with a previous history of any major psychiatric disorder, including psychotic, affective and schizoaffective disorders; head trauma; substance use disorder; or neurological disorders. All participants were followed up for at least 6 months to ensure the diagnosis. Twenty-one out of 60 participants with EOS were naive to drug treatment at the time of MRI scanning, and the remaining 39 had been treated with second-generation antipsychotics at a low dosage (average daily dose equivalent of 5.32 mg olanzapine). Of the 39 treated individuals, 27 had taken drugs for less than a week, 9 for a week to a month, and 3 for one to three months.

Healthy controls (*n* = 44) were recruited from ordinary primary/secondary schools in Chengdu. They were screened by the Mini International Neuropsychiatric Interview for Children and Adolescents (MINI-Kid) to exclude psychiatric disorders. Subjects were excluded if a first/second/third-degree relative suffered from any mental disorders. Four HCs were excluded due to poor-quality MRI. All participants were right-handed (Annett Handedness Scale (26)).

A family history of schizophrenia was obtained by interviewing each participant, both parents, and other first-degree relatives where possible; all interviewees provided detailed information on family history during the clinical interview. This study adopted the definition of family history as described by Xu et al. (27). Familial early-onset schizophrenia (FEOS) was defined as having at least one relative with schizophrenia among their first-, second- or third-degree relatives; otherwise, they were defined as sporadic early-onset schizophrenia (SEOS). Within the FEOS group (*n* = 11), 5 had first-degree relatives, 5 had second-degree relatives, and the other had third-degree relatives with a history of schizophrenia.

Written informed consent was obtained from the parents and the subjects with consenting capacity. This study complied with the content and requirements of the Helsinki Declaration and was reviewed and approved by the Medical Ethics Committee of West China Hospital of Sichuan University.

### MRI Scans

All participants underwent MRI scanning in the Department of Radiology at West China Hospital using a Signa 3.0 T scanner (Achieva, Philips, Netherlands). Foam padding and earplugs were used to reduce head movement and scanner noise. A number of pulse sequences [T2-weighted and two-dimensional (2D), fluid-attenuated inversion recovery (FLAIR)] and image contrasts were collected for clinical review. T1w images were acquired by a magnetization-prepared rapid-acquisition gradient-echo (MPRAGE) sequence: repetition time (TR): 8.1 ms, echo time (TE): 3.7 ms, inversion time (TI): 1072.4 ms, flip angle: 7°, slice thickness: 1 mm (no slice gap), 188 axial slices, matrix size: 256 × 256, field of view (FOV): 256 × 256 mm, and voxel size: 1 × 1 × 1 mm. Slice orientation:sagittal, the phase encode directions:anterior to posterior.T2w images were acquired by a turbo spin-echo sequence: TR: 2500 ms, TE: 261 ms, f lip angle: 90°, slice thickness: 1 mm (no slice gap), 180 axial slices, matrix size: 256 × 256, FOV: 256 × 256 mm, voxel size: 1 × 1 × 1 mm, with strong fat suppression.

### Image Processing: Voxel-Based Morphometry

Image files in DICOM format were transformed to NIfTI format using MRI Convert software^1^. The 3D T1-weighted images were processed using voxel-based morphometry-diffeomorphic anatomical registration through exponentiated Lie algebra (VBM-DARTEL) in SPM12^2^ software and run on the MATLAB (R2017a) platform. The preprocessing steps were as follows: (1) Coordinates: The position of the slice passing through the anterior commissure and posterior commissure was defined as zero; (2) New segment: GM was automatically segmented using tissue signal intensity values or tissue priors for the distribution of brain tissue type (such as gray matter, white matter and cerebrospinal fluid), and GM/white matter images were averaged automatically; (3) Run DARTEL (Create Templates): using the average image as the initial template, the GM images of the subjects were registered with the template, and then the images were averaged to obtain the template for the next iteration. This process was repeated until an optimal template was obtained; (4) Normalize to Montreal Neurological Institute (MNI) space: performing an affine transformation of segmented brain maps into the MNI space(modulation was performed); and (5) Smooth: images were smoothed with an 8 mm × 8 mm × 8 mm full width at half maximum (FWHM) Gaussian kernel.

### Image Processing: FreeSurfer

FreeSurfer’s (v6.0)^3^ standard automatic reconstruction algorithm was used to segment GM/white matter (WM) and reconstruct cortical surfaces. The preprocessing steps included normalization of tissue intensity heterogeneity, removal of non-brain tissue, and segmentation of GM/WM tissue. Each image was carefully inspected, and any segmentation errors were manually corrected by a trained investigator who was blinded to the subject groups. Then, the segmentation calculation was performed again, and the cortex was reorganized by registration with a standard brain template. After reconstruction, it was registered on the sphere template (Fsaverage template) and smoothed with a 10 mm × 10 mm × 10 mm FWHM Gaussian kernel. The Fsaverage Template was used because previous work has found it is suitable for the age range of young samples (28, 29).

### Statistical Analyzes

Statistical analysis was performed with the Statistical Package for the Social Sciences (SPSS 22.0 for Windows, IBM Corp., Armonk, NY, United States). Chi-square tests, Student’s *t*-tests and analysis of variance (ANOVA) were used to compare the distribution and differences of categorical and continuous data, respectively. Mann-Whitney U test was used to compare the difference of disease course, medication time, equal effective dose of olanzapine between FEOS and SEOS group.

First, the comparison of GMV between the EOS and HCs was performed by using two-sample *t*-tests on the statistical parametric maps with sex, age, and total brain volume as covariates. Then, GMV was compared among the FEOS, SEOS, and HC groups by using the analysis of ANOVA, with sex, age, and total brain volume as covariates. Each individual cluster that showed significant differences among groups was defined as a region of interest (ROI). The ROI then was used as explicit mask to compared between groups by using two-sample *t*-tests. We set the significant differences at the threshold of *p* < 0.001 at the voxel level and l*p* < 0.05 at a FDR corrected cluster level.

Second, we used FreeSurfer’s general linear model to compare cortical thickness and surface area between people with EOS and HCs, with sex and age as covariates. Then cortical thickness and surface area were compared among the FEOS, SEOS, and HC groups by using the analysis of ANOVA, with sex, and age as covariates. The difference was statistically significant when *p* < 0.001 at the vertex level and *p* < 0.05 at the cluster level after family wise error (FWE) correction.

Each individual cluster that showed significant differences between groups was defined as a region of interest (ROI). The GMV/cortical thickness of individual ROIs was extracted from each subject. We used Spearman’s rho to explore the association between symptoms (i.e., PANSS subscores) and the values of ROIs. Given that we conducted 3 groups comparisons for each hemisphere (0.005 < 0.05/6), we employed the *p* < 0.005 threshold for the correlational analyzes to control for type II errors in these analyzes (12 tests in 2 groups for each hemisphere).

## Results

### Demographic Characteristics

The demographic characteristics of the participants are shown in Table 1 (EOS and HCs) and Table 2 (FEOS, SEOS, and HCs). There were no significant differences in age (range = 10–16 years; *T* = 1.862, *p* = 0.067), sex (*x*^2^ = 2.232, *p* = 0.135) or education (*T* = 1.24, *p* = 0.219) between people with EOS and HCs. Significant differences were found in education among the FEOS, SEOS and control groups (*F* = 3.726, *p* = 0.02). *Post hoc* analysis found that the SEOS group had significantly higher education than the control group; no significant differences were found in age (*F* = 2.085, *p* = 0.13) or sex (*x*^2^ = 4.36, *p* = 0.113) among these groups. No significant difference was found in age of onset, disease course, medication time, olanzapine equivalent dose or PANSS score between the FEOS and SEOS groups.

**TABLE 1:** Demographic profile of early-onset schizophrenia (EOS) and healthy controls (HCs) [values are mean (S.D.)].

	EOS (*n* = 60)	HCs (*n* = 40)	T/χ 2	*P*
Age, year	14.27 ± 1.471	13.60 ± 1.191	1.862	0.067^a^
Sex (male/female)	21/39	20/20	2.232	0.135^b^
Educational attainment, year	8.13 ± 1.523	7.68 ± 1.979	1.24	0.219^a^
Handedness (left/right)	0/60	0/40	/	/
Age of onset, year	13.75 ± 1.612	/	/	/
Disease course, month	5.433 ± 7.883	/	/	/
Medication time, day	8.45 ± 19	/	/	/
Equal effective dose of olanzapine, mg	5.32 ± 5.94	/	/	/
Score of positive symptoms scale	21.43 ± 6.07	/	/	/
Score of negative symptoms scale	19.92 ± 8.12	/	/	/
General Psychopathology Scale	37.83 ± 12.51	/	/	/
PANSS	79.18 ± 23.98	/	/	/

*EOS: Early-onset schizophrenia; HCs: Healthy controls.*

*^a^Two sample T test.*

*^b^Chi-square test.*

*p < 0.05.*

**TABLE 2:** Demographic profile of familial early-onset schizophrenia (FEOS), sporadic early-onset schizophrenia (SEOS) and healthy controls (HCs) [values are mean (S.D.)].

	FEOS (*n* = 11)	SEOS (*n* = 49)	HCs (*n* = 40)	T/F	*P*
Age, year	14 ± 1.265	14.33 ± 1.519	13.6 ± 1.919	2.085	0.13^a^
Sex (male/female)	6/5	15/34	20/20	4.36	0.113^a^
Education, year	7.91 ± 1.375	8.67 ± 1.625	7.68 ± 1.979	3.726	0.02^a^
Handness (left/right)	0/11	0/49	0/40	/	/
Age of onset, year	13.73 ± 1.191	13.76 ± 1.702	/	−0.51	0.959^b^
Disease course, month	3.91 ± 5.108	5.78 ± 8.385	/	/	0.677^c^
Medication time, day	4.18 ± 8.931	9.4 ± 20.758	/	/	0.183^c^
Equal effective dose of olanzapine, mg	4.51 ± 5.76	5.49 ± 6.02	/	/	0.584^c^
Score of positive symptoms scale	21.27 ± 4.45	21.47 ± 6.42	/	−0.09	0.924^b^
Score of negative symptoms scale	22.9 ± 7.73	19.24 ± 8.11	/	1.407	0.18^b^
General Psychopathology Scale	40.27 ± 13.09	37.28 ± 12.44	/	0.69	0.501^b^
PANSS	84.45 ± 23.22	78 ± 23	/	0.834	0.417^b^

*FEOS: Familial early-onset schizophrenia; SEOS: Sporadic early-onset schizophrenia; HCs: Healthy controls.*

*^a^Chi-square test.*

*^b^Two sample T test.*

*^c^Mann-Whitney U.*

*P < 0.05.*

### Comparison Between the Early-Onset Schizophrenia and Healthy Control Groups

**FIGURE 1:**
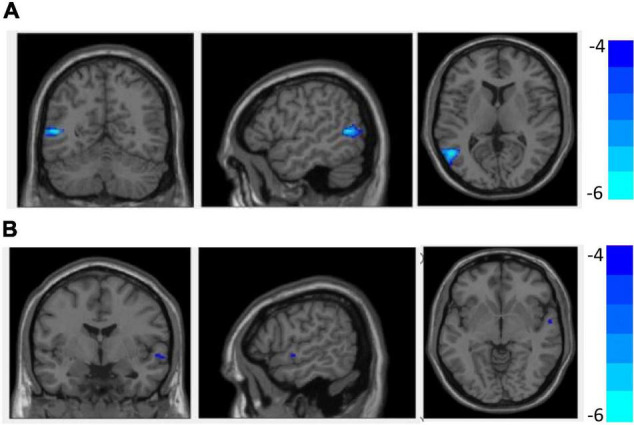
Compared with healthy controls (HCs), grey matter volume (GMV) was decreased in the left middle temporal gyrus **(A)** and right middle temporal gyrus **(B)** in early-onset schizophrenia (EOS) group (*p* < 0.05, FDR corrected).

**FIGURE 2:**
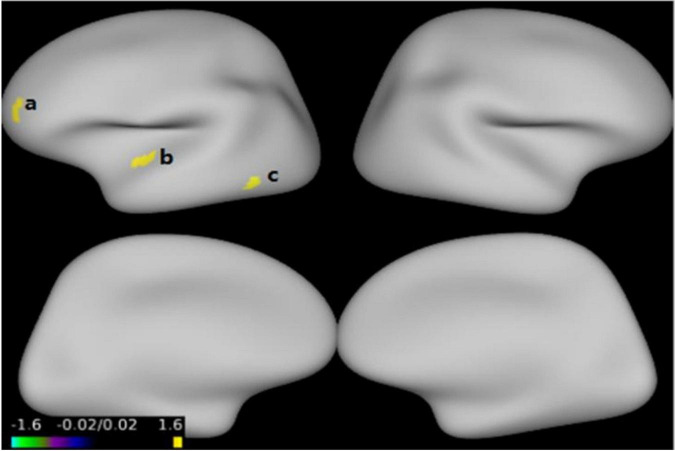
Compared with healthy control (HC) group, cortical thickness was thinner in the left inferior temporal gyrus **(a)**, left superior temporal gyrus **(b)**, left middle frontal gyrus **(c)** in early-onset schizophrenia (EOS) group (*p* < 0.05, FWE corrected).

**FIGURE 3:**
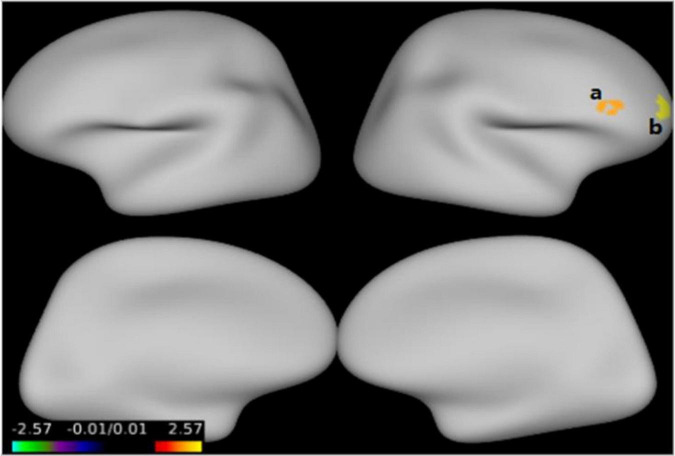
Compared with the healthy controls (HCs), cortical thickness was thinner in the right middle frontal gyrus **(a)**, right inferior frontal gyrus **(b)** in early-onset schizophrenia (EOS) group (*p* < 0.05, FWE corrected).

Figure 1 and Table 3 show that GMV in the EOS group, compared to the control group, was decreased in the left middle temporal gyrus (MTG) (*T* = −5.62, cluster size = 631) and right MTG (*T* = −4.29, cluster size = 31). Figures 2, 3 and Table 4 show that reduced cortical thickness was found in the EOS group in the left inferior temporal gyrus (ITG) (*p* = 0.026, *T* = −3.91), left superior temporal gyrus (STG) (*p* = 0.03, *T* = −3.99), left middle frontal gyrus (MFG) (*p* = 0.03, *T* = −4.07), right MFG (*p* = 0.003, *T* = −3.83), and right inferior frontal gyrus (IFG) (*p* = 0.009, *T* = −3.91). No significant differences in cortical surface area were found between the two groups.

**TABLE 3:** Abnormal grey matter volume (GMV) between the comparison of the groups.

	Cluster size	T	MNI coordinates (X,Y,Z)	Anatomical regions
**EOS < HCs**				
	31	–4.29	58.5, −3, −4.5	Right middle temporal gyrus
	631	–5.62	−52.5, −58.5, 7.5	Left middle temporal gyrus
**SEOS < HCs**				
	282	–5.97	−48, −57, 6	Left middle temporal gyrus

*MNI: The Montreal Neurological Institute; EOS: Early-onset schizophrenia; SEOS: Sporadic early-onset schizophrenia; HCs: Healthy controls.*

*p < 0.05, FDR corrected.*

**TABLE 4:** Reduced cortical thickness between the comparison of the early-onset schizophrenia (EOS) and healthy controls (HCs) groups.

	Cluster no.	Cluster-wise *p*	T	MNI coordinates (X,Y,Z)	Anatomical regions
**EOS < HCs**					
	1	0.026	–3.91	−52, −60, −6	Left inferior temporal gyrus
	2	0.03	–3.99	−49, −15, −5	Left superior temporal gyrus
	3	0.03	–4.07	−29, 48, 5	Left middle frontal gyrus
	4	0.003	–3.83	31, 50, 1	Right middle frontal gyrus
	5	0.009	–3.91	38, 33, 14	Right inferior frontal gyrus

*MNI: The Montreal Neurological Institute; EOS: Early-onset schizophrenia; HCs: Healthy controls.*

*p < 0.05, FWE corrected.*

### Comparison Between the Familial Early-Onset Schizophrenia, Sporadic Early-Onset Schizophrenia and Control Groups

**FIGURE 4:**
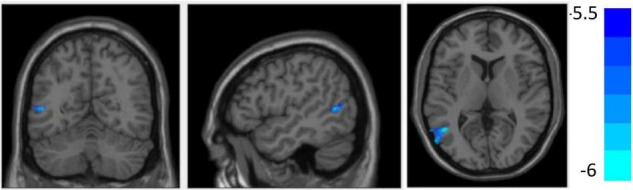
Compared with the healthy controls (HCs), grey matter volume (GMV) reduced in the left middle temporal gyrus in sporadic early-onset schizophrenia (SEOS) group (*P* < 0.05, FDR corrected).

Figure 4 and Table 3 show that compared with the HC group, decreased GMV was found in the SEOS group in the left MTG (*T* = −5.97, cluster size = 282) (*p* < 0.05, FDR corrected). No significant difference of cortical thickness nor surface area was found among the three groups.

### Correlation Between the Region of Interest and Clinical Measures

There was no significant correlation between values of ROIs and PANSS total/subscale scores in the EOS group. There was no significant correlation between values of ROIs and PANSS total/subscale scores in the FEOS nor SEOS group.

## Discussion

The combined VBM and SBM analysis revealed significantly decreased GMV in the bilateral MTG and reduced cortical thickness in the left ITG, STG, MFG and right MFG, IFG in the EOS group. The analysis of the impact of family history showed that the FEOS and SEOS group showed different brain structure morphology.

### Comparison of Grey Matter Volume, Cortical Thickness and Surface Area Between the Early-Onset Schizophrenia and Healthy Control Groups

In line with previous studies, this study supports the finding that individuals with EOS showed decreased GMV in the bilateral MTG (30, 31). Some studies also found decreased GMV in the bilateral MTG in young first-degree relatives of people with schizophrenia (32). A similar pattern was found in both AOS and their healthy siblings (33, 34). The MTG is associated with cognitive functions such as semantic memory encoding, observational movement, deductive reasoning and advanced sensory processing (35, 36). As one of the key regions in the social brain network, the MTG has been widely reported to be associated with schizophrenia and other childhood-onset mental disorders, such as autism spectrum disorder (37–39). Decreased GMV in the MTG among individuals with EOS is consistent with schizophrenia being a disorder of neurodevelopment. This is because the MTG is reported to be a phylogenetically late-developing region, and it has no homolog in non-human primates (40, 41). Therefore, the MTG can express a high degree of interindividual variability in morphology, which is caused by differences in neurodevelopmental processes such as intra- and interareal connections of nerve cells, synaptic development, neuronal migration and differentiation, and cytoarchitectonic formation (38). Decreased GMV observed in the EOS extends the observations from adults, which suggests that the reduction in GMV in the bilateral MTG may be a stable biomarker in both early-onset and adult-onset schizophrenia.

Consistent with previous research, we found reduced cortical thickness in the left ITG, STG, MFG, right MFG, and IFG in the EOS group (42, 43). Although some studies did not find regions with greater/less cortical thickness in EOS (44, 45), there have been a large number of consistent reports on cortical thinning in the EOS group (13, 46, 47). Brain regions such as the frontal and temporal lobes were most commonly reported.

The frontal and temporal lobes are thought to be associated with cognitive functions such as visual processing, language, emotional processing, executive function and decision-making in people with schizophrenia (48). Cortical thickness changes in these brain areas may be associated with abnormal behavior in schizophrenia (49). Two meta-regression analyzes showed that a common pattern of thinning of GM in the left lateral temporal lobe in schizophrenia was significantly associated with positive symptoms and aggression (50). The frontal and temporal lobes are related to higher functions such as cognition, speech, thinking and emotion and are relatively late to mature (40). The brain undergoes dramatic changes during adolescence, with the elimination of millions of synapses and their associated neuronal processes (dendrites and axon terminals) (51). Changes in GMV and cortical thickness in the frontal and temporal lobes in individuals with EOS may be associated with abnormal synaptic pruning in these areas. It has been suggested that schizophrenia occurs with the dysfunction of healthy brain maturation during adolescence (52), which may be due to abnormalities in the genes coding for these trophic factors (53). This may explain the finding of structural brain abnormalities in the EOS group. Compared with AOS, EOS show more problems with thinking disorder, emotional poverty, and cognitive dysfunction. Brain structure changes mainly in the frontal and temporal lobes in the study may be consistent with such prominent symptoms in EOS. Reduced cortical thickness in the frontal and temporal lobes may be considered to be a fundamental pathological feature of EOS.

However, we found no significant differences in surface area between the two groups. Using FreeSurfer, Janssen et al. also reported that there were no significant differences in brain surface area between the EOS and HC groups (13). Cortical surface area is formed by symmetrical division of cortical progenitor cells in ventricular and subventricular regions and increases rapidly due to the curling and folding of the cortex. Unlike cortical thickness, which is associated with changes in neuronal dendrites, dendritic spines, and myelin sheaths in specific brain regions, cortical surface area is related only to the size of neurons and is not affected by brain maturity (54). The results indicated that the abnormal brain structure of individuals with EOS is not associated with cortical surface area.

We found decreased GMV and reduced cortical thickness in the EOS group. However, no significant differences in surface area were found in the EOS group. The abnormal brain regions identified by the two analysis methods were inconsistent, and further analyzes are needed to investigate associations between GMV and cortical thickness. In other words, the differences highlight the complementary effects of the combined analysis of the three indexes. If only one index is used, the characteristics of the lesions cannot be more comprehensively understood, and the causes of the lesions cannot be independently assessed. The results show that it is necessary to simultaneously analyze the three indexes in future studies.

### Comparison of Grey Matter Volume, Cortical Thickness and Surface Area Between Subgroups

To the best of our knowledge, this is the first study to conduct an exploratory analysis of whether the presence of a family history of schizophrenia causes differences in brain structure in EOS.

The onset of EOS is closely related to environmental factors (55). Environmental factors such as parents? reproductive age, obstetric complications, childbirth season, behavioral biases or language retardation, exposure to adverse life events and drug use can all increase the risk of EOS (56–59). The above environmental factors are likely to induce molecular genetic changes, which may cause damage to the brain structure in those with SSP. Although we did not find differences between the FEOS and SEOS groups, we found decreased GMV in the SEOS group in the left MTG when compared with the HCs group. Our earlier studies in AOS reported the WM fiber bundles were more severely damaged in the SSP group than the FSP group (21). Using FreeSurfer, we found a decreased surface area in the left prefrontal lobe in the SSP group compared with the FSP and HC groups (22). These results suggesting that the two types of schizophrenia may have different pathogeneses, and showing a trend that brain changes in sporadic schizophrenia may be more pronounced than in familiar schizophrenia.

The genetic mutations play an important role in those with sporadic schizophrenia. Some phenotypes of people with SSP (such as abnormal brain structure and impaired WM integrity) may be affected by *de novo* copy number (CN) mutations and produce an independent phenotype than that of people with FSP (21, 60). Xu et al. (27) also mentioned that rare germline mutations lead to vulnerability in people with SSP, and rare genetic damage can explain (at least in part) the genetic heterogeneity of schizophrenia at many different genetic loci. However, the proportions of relatives at different levels may have influenced the results. Due to the small number of subjects in the FEOS group, we could not regroup them based on this factor. This study was only an exploratory analysis, and a larger sample size in the FEOS group is needed for further verification.

Our study had three major limitations. First, a small percentage of EOS were treated with antipsychotic drugs, and we cannot exclude the possibility that the drugs may have had an effect on the structure of the brain. However, the medication dosage and duration of administration were relatively small and short, and the medication time, drug dosage and types of drugs between the FEOS and SEOS groups were not significantly different. Second, the small sample size in the FEOS group may have resulted in insufficient power to find differences between subgroups. The results for the subgroup comparisons have to be interpreted with caution. Our findings may be regarded as preliminary and need to be confirmed by a larger sample size in the future. Third, there were no follow-up studies, and the results reflected structural differences in the brain at only one time point.

## Conclusion

We confirmed abnormalities in GMV and cortical thickness, especially in the temporal lobe and frontal lobe, in the early stage of EOS by analyzing a relatively large sample from a Han Chinese population. The FEOS group and SEOS group showed different brain structure morphology.

## Data Availability Statement

The original contributions presented in this study are included in the article/supplementary material, further inquiries can be directed to the corresponding author.

## Ethics Statement

The studies involving human participants were reviewed and approved by Medical Ethics Committee of West China Hospital of Sichuan University. Written informed consent to participate in this study was provided by the participants’ legal guardian/next of kin.

## Author Contributions

JC, TL, and PS: designed the study. JC, WW, LZ, ML, XL, SL, WD, XD, QW, W-jG, and XM: recruited the participants, administered the assessment, and carried out data analysis. JC and WW: wrote the manuscript. TL: revised the manuscript. All authors contributed to the article and approved the submitted version.

## Conflict of Interest

The authors declare that the research was conducted in the absence of any commercial or financial relationships that could be construed as a potential conflict of interest.

## Publisher’s Note

All claims expressed in this article are solely those of the authors and do not necessarily represent those of their affiliated organizations, or those of the publisher, the editors and the reviewers. Any product that may be evaluated in this article, or claim that may be made by its manufacturer, is not guaranteed or endorsed by the publisher.

## Footnotes

1
https://lcni.uoregon.edu/downloads

2
http://www.fil.ion.ucl.ac.uk/spm

3
https://surfer.nmr.mgh.harvard.edu/fswiki/FreeSurferWiki
